# *Aspergillus* leaf spot of field bindweed (*Convolvulus arvensis* L.) caused by *Aspergillus niger* in China

**DOI:** 10.1186/s40064-016-2292-4

**Published:** 2016-05-12

**Authors:** Xuekun Zhang, Hui Xi, Kejian Lin, Zheng Liu, Yu Yu, Yan Sun, Jing Zhao

**Affiliations:** Northwest Inland Region Key Laboratory of Cotton Biology and Genetic Breeding (Xinjiang), Ministry of Agriculture, Institute of Plant Protection, Xinjiang Academy Agricultural and Reclamation Sciences, No. 221 Wuyi Road, Shihezi, China; Institute of Cotton, Shihezi Academy of Agricultural Sciences, Shimo Road, Shihezi, China

**Keywords:** Leaf spot, Field bindweed, Internal transcribed spacer rDNA, β-Tubulin gene, *Aspergillus niger*

## Abstract

Leaf spot was found on field bindweed (*Convolvulus arvensis* L.) in Shihezi City, Xinjiang Province, China, during the summer of 2015. Pathogens were isolated from the infected leaves of field bindweed and identified as *Aspergillus niger* based on morphological and molecular analyses (internal transcribed spacer rDNA and β-tubulin gene). A pathogenicity test confirmed that *Aspergillus niger* caused the healthy leaves of field bindweed to become diseased. To our knowledge, this is the first report of field bindweed infected naturally by *A. niger*.

## Background

Field bindweed is a global malignant perennial weed (Vasilakoglou et al. 2013), which competes with many crops, including corn, wheat, beans, cotton, and vegetables, for water, inorganic salt, nutrients, and light (Rodríguez-Navarro et al. 2011; Vasilakoglou et al. 2013). The qualities and yields of the crops declined in areas where field bindweed occurred (Lindenmayer et al. 2013). Field bindweed has become one of the most serious weed problems in Xinjiang, majorly affecting the growth of cotton and lacking effective methods to control it currently (Ma et al. 2010).

Field bindweed has a natural resistance to chemical herbicides; therefore, conventional herbicides are generally ineffective for its control (Westwood and Weller 1997). Since 1979, plant pathogens have been used as weed management because they were relatively safe and did not result in herbicide resistance (Charudattan and Walker 1982; Aneja et al. 2013). Field bindweed could be naturally infected by *Alternaria triticina* and *Phomopsis longicolla* (Saleem et al. 2015; Li et al. 2010). During the summer of 2015, naturally diseased leaves of field bindweed were collected in Shihezi City. The aims of the present study were to identify the species isolated from field bindweed by using morphological and molecular analyses and provide potential biocontrol resources for field bindweed.

## Methods

### Isolation and cultivation of the pathogen

Field bindweed leaves (2 × 6 mm^2^) were selected at the junction of diseased and healthy tissues. The surface of the selected leaves were sterilized with 0.1 % HgCl_2_ for 45 s and rinsed in sterile distilled water five times after rinsing in 70 % ethanol for 30 s. Thereafter, the chosen leaves were placed on potato dextrose agar (PDA) plates and incubated at 25 °C in a constant temperature unit.

### Identification of the pathogen

The mycological characteristics of the colony, conidiophores, and conidia were observed under a light microscope. Amplification and sequencing of the internal transcribed spacer (ITS) rDNA and β-tubulin gene were used to identify the isolates (Kwon et al. 2012; Choudhury et al. 2014). The results of sequencing were blasted in the GenBank database. The sequences of related species (*Alternaria solani*, *Aspergillus flavus*, *Aspergillus niger*, *Aspergillus ochraceus*, *Aspergillus sydowii*, *Fusarium poae*) were chosen from GenBank and aligned using Clustal X 1.81 software. A phylogenetic tree was constructed with the evolutionary distance data calculated using Kimura’s two-parameter model by using the neighbor-joining method with 1000 bootstrap replicates, by using DNAman software package version 5.2.2.

### Pathogenicity test

The pathogenicity was tested using in vitro and in vivo wounded inoculations. The healthy leaves of field bindweed and flamed needles were used for inoculation. For the in vitro experiment, six sterilized (70 % ethanol) leaves of field bindweed were inoculated with PDA plugs (78.5 mm^2^) of each isolate of a mycelial culture, and non-colonized PDA plugs were used as controls. The petioles of the leaves were wrapped with sterilized pledget, which was soaked in sterile distilled water for 5 s and then placed in sterile Petri dish. For the in vivo experiment, six leaves were inoculated with the spore suspension (10^6^ CFU mL^−1^), and sterile distilled water was used as the control. All the leaves and plants inoculated were placed in a plant growth chamber at 28 °C and 70 % relative humidity.

## Results and discussion

**Fig. 1:**
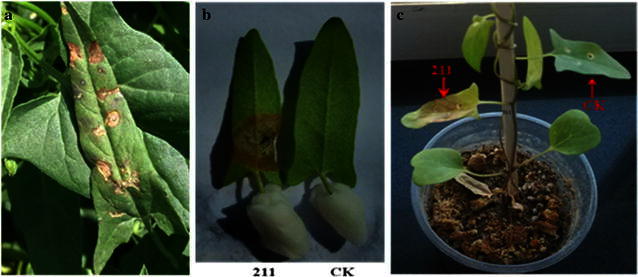
Pathogenicity test to field bindweed by *Aspergillus niger* strain 211. **a** Diseased naturally leaf of field bindweed in the field; **b** wounded inoculation using strain 211 in vitro test; **c** wounded inoculation using strain 211 in vivo test; CK: Control

**Fig. 2:**
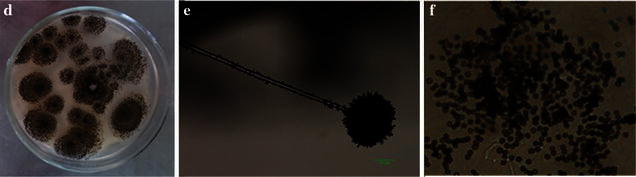
*Aspergillus niger* morphological characteristics of colony, conidiophores and conidia. *Note* **d** The colony of *Aspergillus niger* on PDA; **e** Conidiophores of *Aspergillus niger*; **f** Conidia of *Aspergillus niger*

**Fig. 3:**
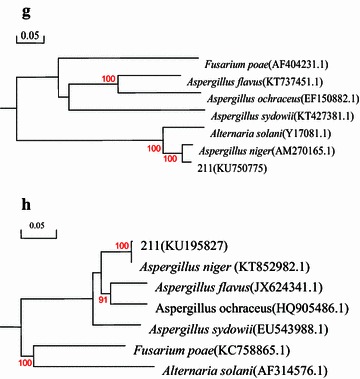
Phylogenetic relationships among *Aspergillus niger* isolates generated through N-J analysis of β-tubulin gene (**g**) and internal transcribed spacer (ITS) rDNA (**h**) sequences using Kimura’s two-parameter models. Bootstrap support values higher than 50 % from 1000 replicates are shown at the nodes

The symptoms of diseased leaves of field bindweed from cotton fields were observed as multiple dark brown spots (Fig. 1a), but the leaves of nearby cotton plants didn’t appear to have similar symptoms. The isolates were identified as *Aspergillus* sp. based on their morphological identification (Lu 2001). The colonies were white originally, which grew fast and appeared as black dots without producing pigment on the PDA 3 days later (Fig. 2d). The conidiophores were 600–3000 μm lengths and 7–21 μm widths, which grew upright, with a swollen top similar to a spherosome (54–140 × 60–141 μm), containing many oval conidia (2.5–3.8 μm in diameter) (Fig. 2e, f). The results of the gene sequences were deposited in GenBank (ITS rDNA accession no. KU195827, β-tubulin gene accession no. KU750775). The molecular identification of isolates was verified by high sequence similarity of both gene sequences of isolate strain 211 with *A. niger* (Fig. 3).

During the pathogenicity test, spots appeared rapidly on leaf surfaces and necrosis appeared on the whole leaf within three to 4 days, whereas the control did not show any symptoms (Fig. 1b, c). Some mycelium appeared on the surface of leaves inoculated in the in vitro but not the in vivo test, because the airtight conditions of the Petri dish caused the humidity in the in vitro test to be higher than that in the in vivo test (Fig. 1b). The inoculated pathogen was re-isolated and the morphology was found to be similar to that of the original isolate.

## Conclusions

Based on the research results, the isolate causing diseased leaves in field bindweed was *A. niger*. Although a previous study of *A. niger* showed that it caused fruit rot of grapes in Xinjiang Province (Zhang et al. 2010), the metabolites showed antifungal activity and could be used to control tomato root-knot nematodes in China (Zhang 2015; Li et al. 2011). To our knowledge, this is the first report of field bindweed naturally infected with *A. niger*. In our future studies, we hope to determine host specificity and security evaluation whether *A. niger* and mycotoxins can be used as effective bioherbicides.
